# Transcriptional upregulation of *Bag3*, a chaperone-assisted selective autophagy factor, in animal models of KY-deficient hereditary myopathy

**DOI:** 10.1242/dmm.033225

**Published:** 2018-07-06

**Authors:** Elliot J. Jokl, Gideon L. Hughes, Tobias Cracknell, Mary E. Pownall, Gonzalo Blanco

**Affiliations:** Department of Biology, University of York, Wentworth Way, York YO10 5DD, UK

**Keywords:** Kyphoscoliosis peptidase, Chaperone-assisted selective autophagy, BAG3, FLNC, Myopathy

## Abstract

The importance of kyphoscoliosis peptidase (KY) in skeletal muscle physiology has recently been emphasised by the identification of novel human myopathies associated with KY deficiency. Neither the pathogenic mechanism of KY deficiency nor a specific role for KY in muscle function have been established. However, aberrant localisation of filamin C (FLNC) in muscle fibres has been shown in humans and mice with loss-of-function mutations in the *KY* gene. FLNC turnover has been proposed to be controlled by chaperone-assisted selective autophagy (CASA), a client-specific and tension-induced pathway that is required for muscle maintenance. Here, we have generated new C2C12 myoblast and zebrafish models of KY deficiency by CRISPR/Cas9 mutagenesis. To obtain insights into the pathogenic mechanism caused by KY deficiency, expression of the co-chaperone BAG3 and other CASA factors was analyzed in the cellular, zebrafish and *ky/ky* mouse models. *Ky*-deficient C2C12-derived clones show trends of higher transcription of CASA factors in differentiated myotubes. The *ky-*deficient zebrafish model (*ky^yo1^/ky^yo1^*) lacks overt signs of pathology, but shows significantly increased *bag3* and *flnca/b* expression in embryos and adult muscle. Additionally, *ky^yo1^/ky^yo1^* embryos challenged by swimming in viscous media show an inability to further increase expression of these factors in contrast with wild-type controls. The *ky/ky* mouse shows elevated expression of *Bag3* in the non-pathological exterior digitorum longus (EDL) and evidence of impaired BAG3 turnover in the pathological soleus. Thus, upregulation of CASA factors appears to be an early and primary molecular hallmark of KY deficiency.

## Abstract (precis)

**Summary:** Increased transcriptional activation of the tension-mediated autophagy factor *Bag3* is shown to be a consistent molecular hallmark of KY deficiency in animal models.

## INTRODUCTION

The first model of kyphoscoliosis peptidase (KY) deficiency was the *ky/ky* mouse, which emerged spontaneously as a model of recessive, hereditary kyphoscoliosis in an inbred mouse strain over 40 years ago (Dickinson and Meikle, 1973; Mason and Palfrey, 1984). Subsequent extensive histopathological analyses showed that postural, tonically active muscles undergo a cycle of degeneration and regeneration, whereas fast-twitch muscles remain relatively unaffected (Bridges et al., 1992). Postural muscles show ultrastructural evidence of sarcomeric damage, including Z-disc thickening and elevated levels of autophagic vacuoles (Bridges et al., 1992). The mutation in the *Ky* gene is a GC deletion near the beginning of the coding sequence that results in a premature stop codon (Blanco et al., 2001); thus, *ky/ky* mice lack any KY protein (Baker et al., 2010). Yeast two-hybrid/biochemical interactions and co-localisation studies in adult fibres led to the proposal of a Z-disc network involving KY, filamin C (FLNC), ZAK and other proteins (Baker et al., 2010; Beatham et al., 2004). Crucially, the *ky/ky* soleus shows aberrant localisation of the cytoskeletal crosslinkers FLNC (Beatham et al., 2004) and XIN (Beatham et al., 2006), which suggests a failure of muscle to maintain its structural integrity under consistent high tension in the absence of KY. Additionally, the muscles of *ky/ky* mice do not undergo compensatory hypertrophy in response to muscle overloading through surgical ablation (Blanco et al., 2001), indicating that KY is necessary to translate increased mechanical tension into muscle growth.

The *Ky* gene encodes a transglutaminase-like protein located at the Z-disc of skeletal muscle (Baker et al., 2010). KY is highly conserved across vertebrates and shares a near-identical protein sequence among mammals. Although the putative catalytic residues are conserved, to date no direct evidence of enzymatic activity over any endogenous substrate has been found. Moreover, alternative alignments suggest that this domain has been co-opted for protein-protein interactions (Anantharaman et al., 2001).

Mutations in the *KY* gene associated with myopathy have been recently reported in human patients (Hedberg-Oldfors et al., 2016; Straussberg et al., 2016; Yogev et al., 2017). Common pathological hallmarks in mice and humans include muscle atrophy of the soleus and the presence of FLNC aggregates. Deficient turnover of FLNC might thus be core to the ‘*ky*’ pathogenic mechanism in humans and mice. FLNC has been established as a client of the chaperone-assisted selective autophagy (CASA) pathway (Ulbricht et al., 2013b). CASA is a recently proposed tension-induced autophagy mechanism (Arndt et al., 2010) that is reliant on the Z-disc co-chaperone BAG3. BAG3 binds to chaperones HSC70 and HSPB8 and facilitates solubilisation of damaged FLNC from the cytoskeleton. FLNC is then ubiquitinated by the E3 ubiquitin ligase CHIP/STUB1 and further complexed with tethering factors for autolysosomal degradation. In addition to mediating the degradation of FLNC, under mechanical tension BAG3 interacts with components of the Hippo signalling network to induce *Flnc* transcription. BAG3 interacts with inhibitors of the YAP/TAZ transcription factors. Once released of its inhibitors, YAP/TAZ translocates to the nucleus and upregulates the synthesis of native FLNC (Ulbricht et al., 2013a). Hence, BAG3 facilitates degradation and synthesis of FLNC, leading to the suggestion that tension-bearing cells adapt FLNC turnover rates to the level of tension (Ulbricht et al., 2013a).

Given that FLNC is a known KY interaction partner (Beatham et al., 2004), it is plausible that its abnormal distribution in KY-deficient mice and humans reflects an alteration of the CASA pathway. Here, we investigate factors involved in CASA in newly generated myotube (C2C12) and zebrafish models of KY deficiency and in *ky/ky* mice. Our results indicate that changes in transcriptional activation of CASA components are an early hallmark of KY deficiency in these models.

## RESULTS

### Elevated expression of CASA components in C2C12-derived *Ky*-knockout myotubes

BAG3 and FLNC expression positively correlate with increasing tension in smooth muscle cells (Ulbricht et al., 2013a). To test whether KY is involved in a similar pathway in skeletal muscle cells, we used CRISPR/Cas9 technology to generate C2C12-derived clones with disruptive mutations in *Ky* (Fig. S1; see Materials and Methods section for details). Quantitative real-time PCR (qPCR) assays showed a significant reduction of *Ky* transcript in clones D, I and K after differentiation into myotubes; either *Hprt* (housekeeping gene) or *Myh7* (differentiation marker; see Fig. S2) were used as controls for normalisation, showing that these differences cannot be accounted for by any varying levels of differentiation between wild-type (WT) and mutant clones (Fig. S1E). This finding is consistent with previous evidence of the nonsense-mediated decay of this transcript found in the mouse *ky* mutant (Blanco et al., 2001). These *Ky*-deficient clones were then selected for further analyses.

**Fig. 1.**
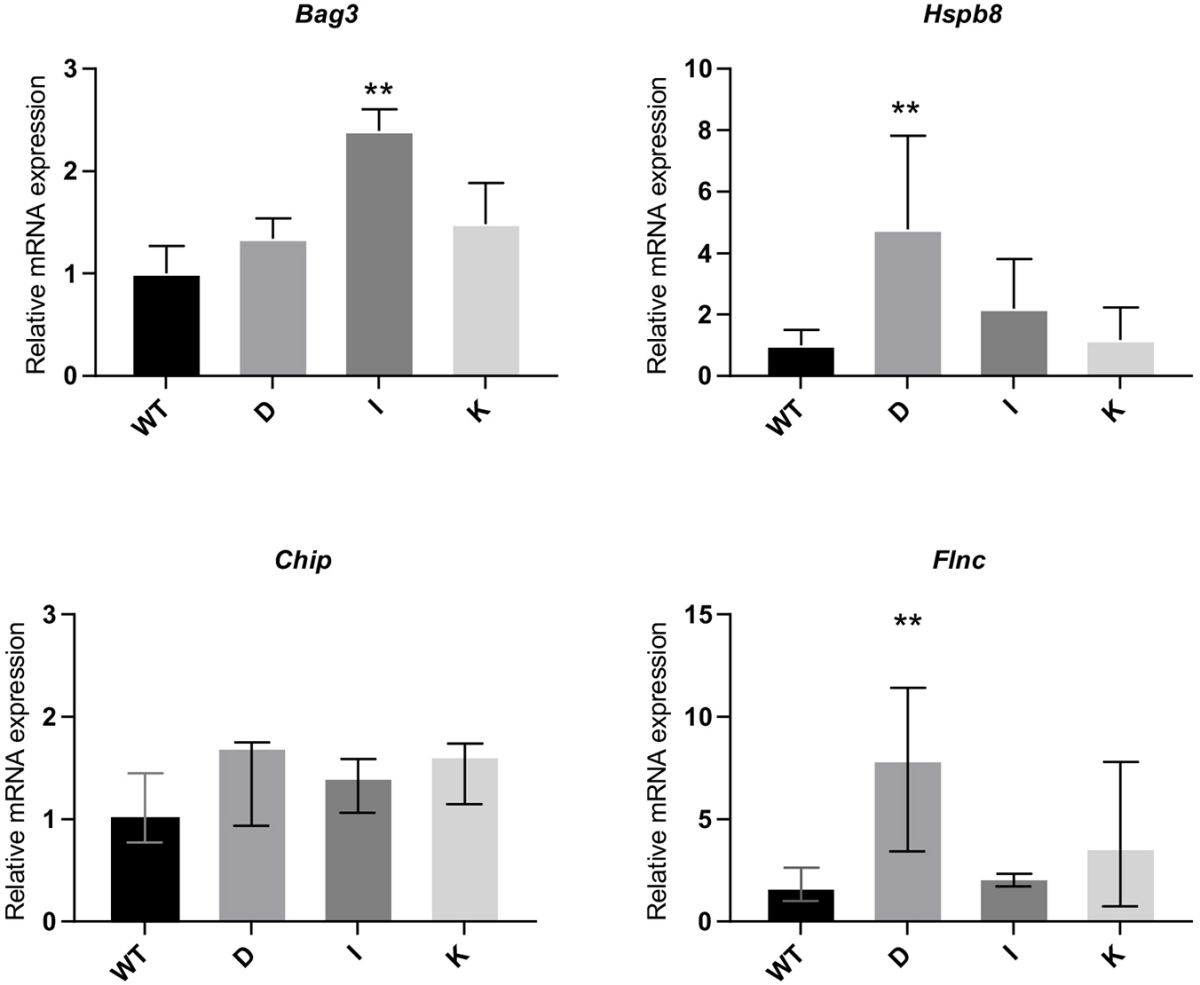
**Transcriptional upregulation of CASA components in *Ky*-deficient differentiated myotubes.** *Chip, Bag3, Hspb8* and *Flnc* qPCR results show consistent upward trends in expression in *Ky-*deficient myotubes relative to WT, reaching significance for CASA components *Bag3, Hspb8* or *Flnc* in two of the *Ky*-deficient clones (one-way ANOVA/Dunnett's for *Bag3 and Hspb8*; Kruskal-Wallace/Dunnett's for *Chip* and *Flnc*; **P<0.01; *n*≥6). Error bars indicate s.e.m. for *Bag3 and Hspb8* and median and interquartile range for *Chip* and *Flnc.*

Given that *Flnc* is only expressed in differentiated cells (Dalkilic et al., 2006) and *Ky* is also highly expressed in these conditions (Baker et al., 2010), the analysis of CASA factors was focused on myotubes. We tested the expression of *Bag3, Hspb8, Chip* and the main CASA client *Flnc*. For qPCR assays, genes that are consistently expressed throughout differentiation were normalised to *Hprt*. However, differentiation in culture, particularly the length of time required for myotube formation, is influenced by a number of factors (e.g. number of passages, flask size and coating). To minimise the effect of the differentiation level as a confounding factor in our measurements of target gene relative expression, we used an indicator of the state of differentiation, *Myh7*, as internal normalisation marker for *Flnc*. qPCR results revealed a consistent trend towards elevated expression of CASA components: all mutant lines showed higher means for all components, reaching significance for *Flnc* (clone D)*, Hspb8* (clone D) and *Bag3* (clone I) (Fig. 1). The inconsistency of the significance of these trends between clones, however, prevents these observations from being conclusive.

### Mutagenesis of *ky* in zebrafish via CRISPR/Cas9 genome editing

**Fig. 2.**
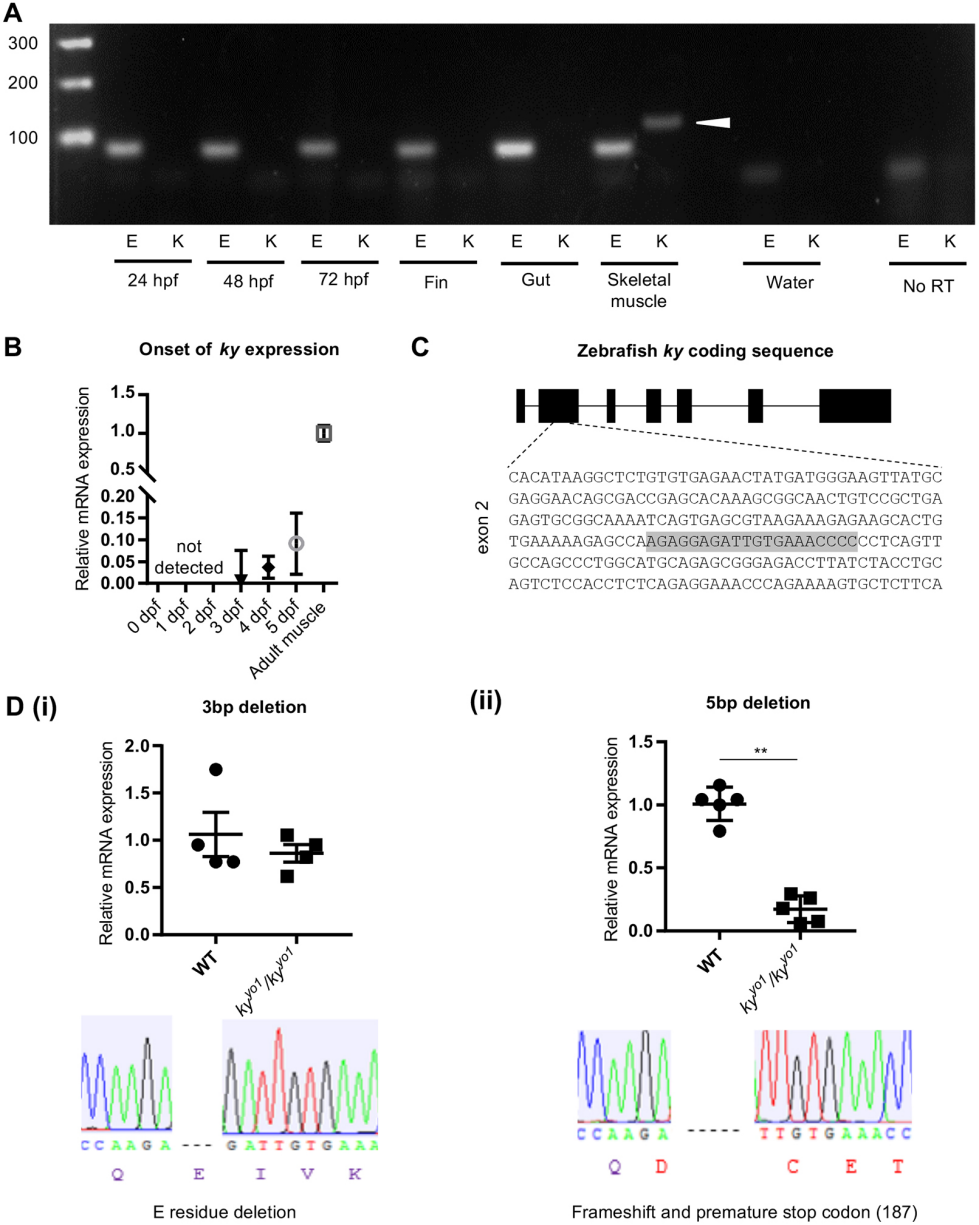
**Generation and validation of a *ky*-knockout zebrafish line *ky^yo1^/ky^yo1^*.** (A) qPCR of the identified *ky* orthologue shows no expression in the early embryonic stages or in adult fin and gut tissue. Robust expression is seen in skeletal muscle (white arrow), consistent with the expression profile reported in mice. Embryonic time points (hpf) and tissues, as indicated. E, positive control *ef1a*; K, *ky*. (B) qPCR shows that the onset of *ky* expression is around 3 dpf, with expression appearing to increase between 3 and 5 dpf. Expression at these stages is much lower than in adult skeletal muscle, but this might partially be accounted for by the relative contribution of muscle-specific transcripts to the whole embryo. Error bars indicate s.d. of three technical replicates. (C) Full exon intron structure of zebrafish *ky* (XM_001335276.5, verified by qPCR) at scale with the CRISPR/Cas9 target sequence highlighted in grey in the exon 2 sequence. (D) qPCR of *ky* shows that fish homozygous for a 3 bp deletion (i), modelled to result in the deletion of a single glutamic acid (E) residue, show no decrease in transcript expression. By contrast, fish homozygous for a 5 bp deletion (ii), modelled to result in a frameshift and premature stop codon, show a significant decrease in transcript expression (Student's *t*-test; *P*<0.01, *n*=3), indicating disruption at the mRNA level presumably by nonsense-mediated decay. Error bars indicate s.e.m.

A zebrafish orthologue of *ky* (Ensembl ENSDARG00000074036) is located on chromosome 13. A tBLASTn query of the zebrafish nucleotide database with the mouse KY protein retrieved only the annotated zebrafish *ky*, indicating that no other putative orthologue exists in the zebrafish genome. Consistent with this, our results using HMMER (Finn et al., 2011), a homology search software designed to detect remote homologues, to query the mouse genome with the putative zebrafish *ky* sequence returned murine KY as the top result in the mouse protein database. Expression of the *ky* orthologue in zebrafish was analysed in the embryo and adult tissues by endpoint qPCR. Expression was detected in adult skeletal muscle, but not in other adult tissues or early embryonic stages (Fig. 2A). qPCR detected expression of *ky* from 3 dpf and higher levels of expression in adult skeletal muscle (Fig. 2B). These results are consistent with the muscle-specific expression previously reported for the mouse *Ky* gene (Blanco et al., 2001).

Gene targeting of zebrafish *ky* was carried out using a guide RNA directed against exon 2 (Fig. 2C), which was generated by an annealed-extended oligo template method (Nakayama et al., 2013). The guide RNA was co-injected with purified Cas9 protein into fertilised embryos at the 1- to 8-cell stages. The efficiency of gene targeting was assayed at 24 hpf using heteroduplex analyses of PCR amplicons from individual embryos, confirming the presence of sequence variants. Chimeric F0 zebrafish were outcrossed to wild-type fish to produce F1 offspring carrying defined heterozygous mutations in *ky.* Heterozygous fish carrying specific mutations were selected for incrosses to produce homozygous mutant fish. A disruptive mutation (5 bp deletion) and a non-disruptive one (3 bp deletion), both occurring at codons 141/142 within exon 2 (Fig. 2D), were used to generate homozygous zebrafish lines for further analyses.

qPCR showed a significant 10-fold reduction in *ky* transcript levels in the 5 bp deletion fish muscle at 3 months compared with WT (Fig. 2D), indicating degradation of mutant transcript by nonsense-mediated decay. No significant difference was observed in the transcript levels of 3 bp deletion and WT (Fig. 2D). The disruptive allele was given the Zebrafish Information Network (ZFIN) designation *ky^yo1^*.

### Upregulation of some CASA components in *ky^yo1^/ky^yo1^* zebrafish

**Fig. 3.**
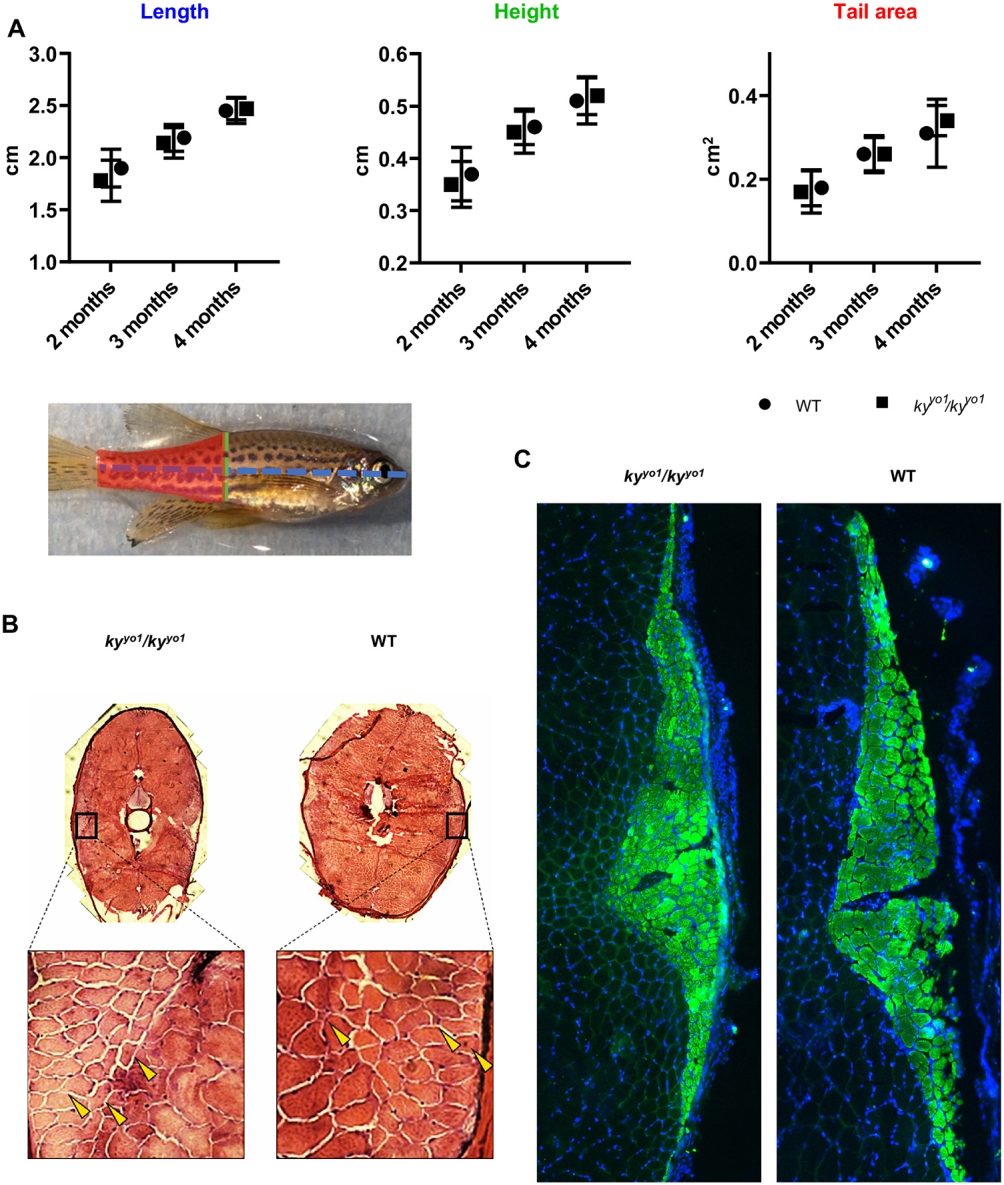
**Organism and tissue-level characterisation of the *ky^yo1^/ky^yo1^* zebrafish shows no overt pathology.** (A) Longitudinal growth study shows no difference in *ky^yo1^/ky^yo1^* zebrafish morphology. Three metrics were assessed in WT and *ky^yo1^/ky^yo1^* zebrafish at 2, 3 and 4 months post-fertilisation: length (blue) from the jaw to the anterior of the tail fin, height (green) at the anterior of the anal fin, and tail area (red) measured between the anterior of the anal fin and the anterior of the tail fin. No differences were seen between WT and *ky^yo1^/ky^yo1^* zebrafish. (B) Representative transverse sections of 3-month-old zebrafish show muscle fibres in cross-section. A small number of very small fibres and fibres with centralised nuclei can be seen with similar frequency in both WT and *ky^yo1^/ky^yo1^* fish (examples indicated by yellow arrows), indicating that these are not pathological changes. (C) Slow muscle distribution is preserved in the *ky^yo1^/ky^yo1^* zebrafish. Immunofluorescence against the slow-muscle marker (s58) on transverse sections shows that slow muscle remains restricted laterally in the *ky^yo1^/ky^yo1^* zebrafish. Image representative of sections from two WT and two *ky^yo1^/ky^yo1^* fish at 6 months post-fertilisation. As sections are derived from different positions on the anterior-posterior axis, slow-muscle area and cell number cannot be directly compared.

**Fig. 4.**
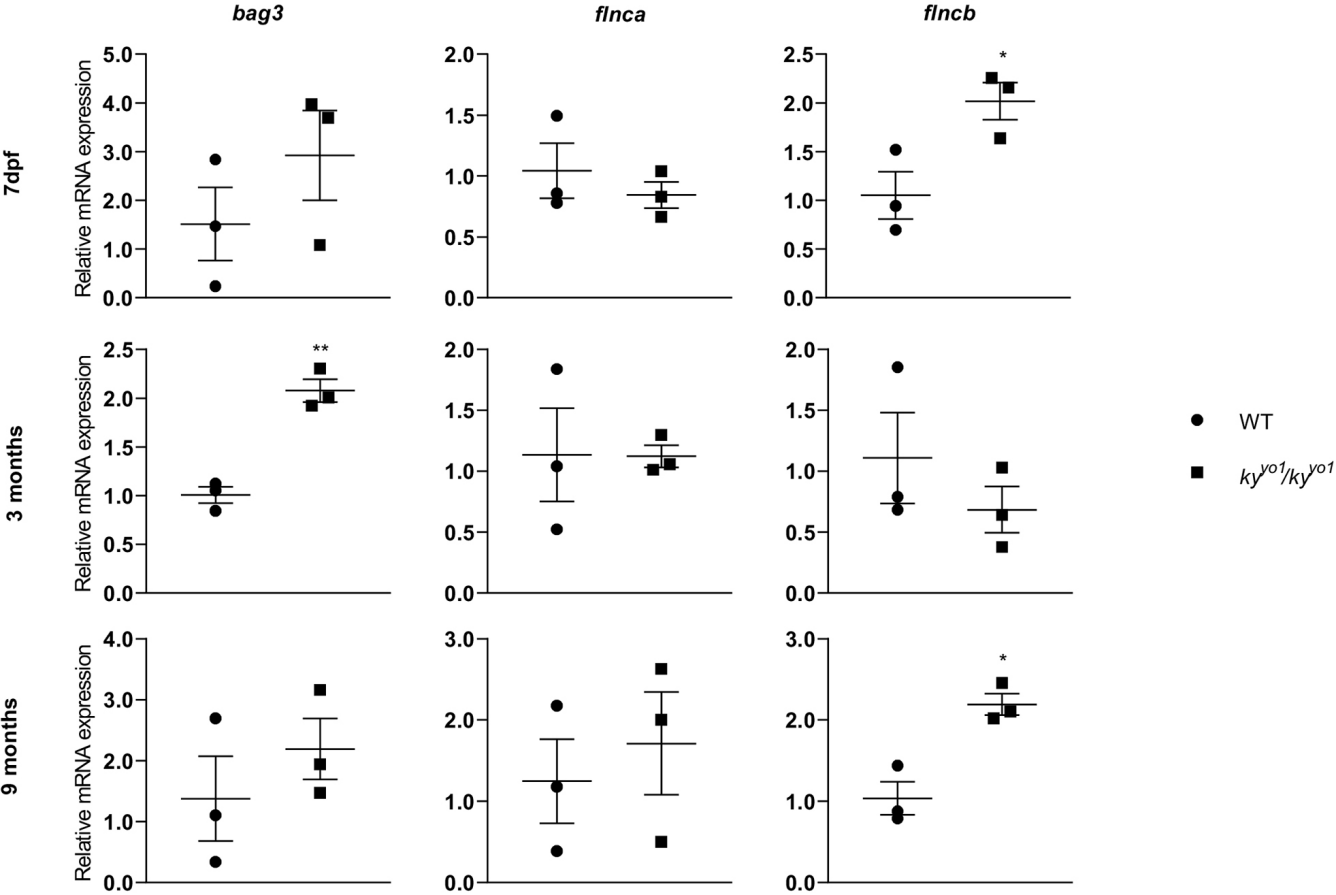
**Transcriptional analysis of CASA components in the *ky^yo1^/ky^yo1^* zebrafish line.** qPCR of *bag3, flnca* and *flncb* at 7 days, 3 months and 9 months post-fertilisation shows a persistent trend of increased *bag3* transcription, although this only achieves significance at the 3 month stage (one-way ANOVA/Dunnett's; *P*<0.01, *n*=3). Levels of *flnca* show no apparent differences and *flncb* shows a significant increase at 7 dpf and 9 months (one-way ANOVA/Dunnett's; *P*<0.05, *n*=3). Error bars indicate s.e.m.

Development of muscle in *ky^yo1^/ky^yo1^* zebrafish embryos is normal, consistent with the late-embryonic onset of *ky* expression, and adults are viable and fertile. No morphological differences were observed in *ky^yo1^/ky^yo1^* juvenile fish (2-4 months old): the length, height at anterior of anal fin (HAA) and tail area (area between HAA line and anterior boundary of the tail fin) showed no difference to those of WT fish (Fig. 3A). Skeletal muscle is morphologically normal on Hematoxylin and Eosin (H&E)-stained cross-sections (Fig. 3B). Given that *ky/ky* mice show a progressive shift towards expression of slower fibre types (Marechal et al., 1996), we analysed the expression of type I fibres in *ky^yo1^/ky^yo1^* juvenile fish muscles by immunofluorescence. Fibre-typing showed that slow muscle remained exclusively in the lateral region of skeletal muscle both in WT and *ky^yo1^/ky^yo1^* fish (Fig. 3C). We conclude that the absence of Ky in zebrafish does not have the same effect on muscle morphology or fibre type as seen in *ky* mutant mice (Blanco et al., 2001) or in patients with KY-associated myopathy (Hedberg-Oldfors et al., 2016). Analysis of the molecular effects of KY deficiency provided some evidence of functional conservation, however. We showed that C2C12-derived myotubes expressed higher levels of CASA factors in the absence of KY (see Fig. 1); therefore, we tested whether the zebrafish orthologues of *bag3* and *flnc* (*flnca* and *flncb*) were similarly regulated by KY. In Fig. 4, we show transcriptional upregulation of *bag3* and *flncb* in *ky^yo1^*/*ky^yo1^* zebrafish. qPCR analysis of *ky^yo1^*/*ky^yo1^* mutant zebrafish compared with wild-type siblings reveals a significant increase of *bag3* (at 3 months) and *flncb* (at 7 dpf and 9 months).

### Embryos lacking Ky show impaired mechanotransduction

**Fig. 5.**
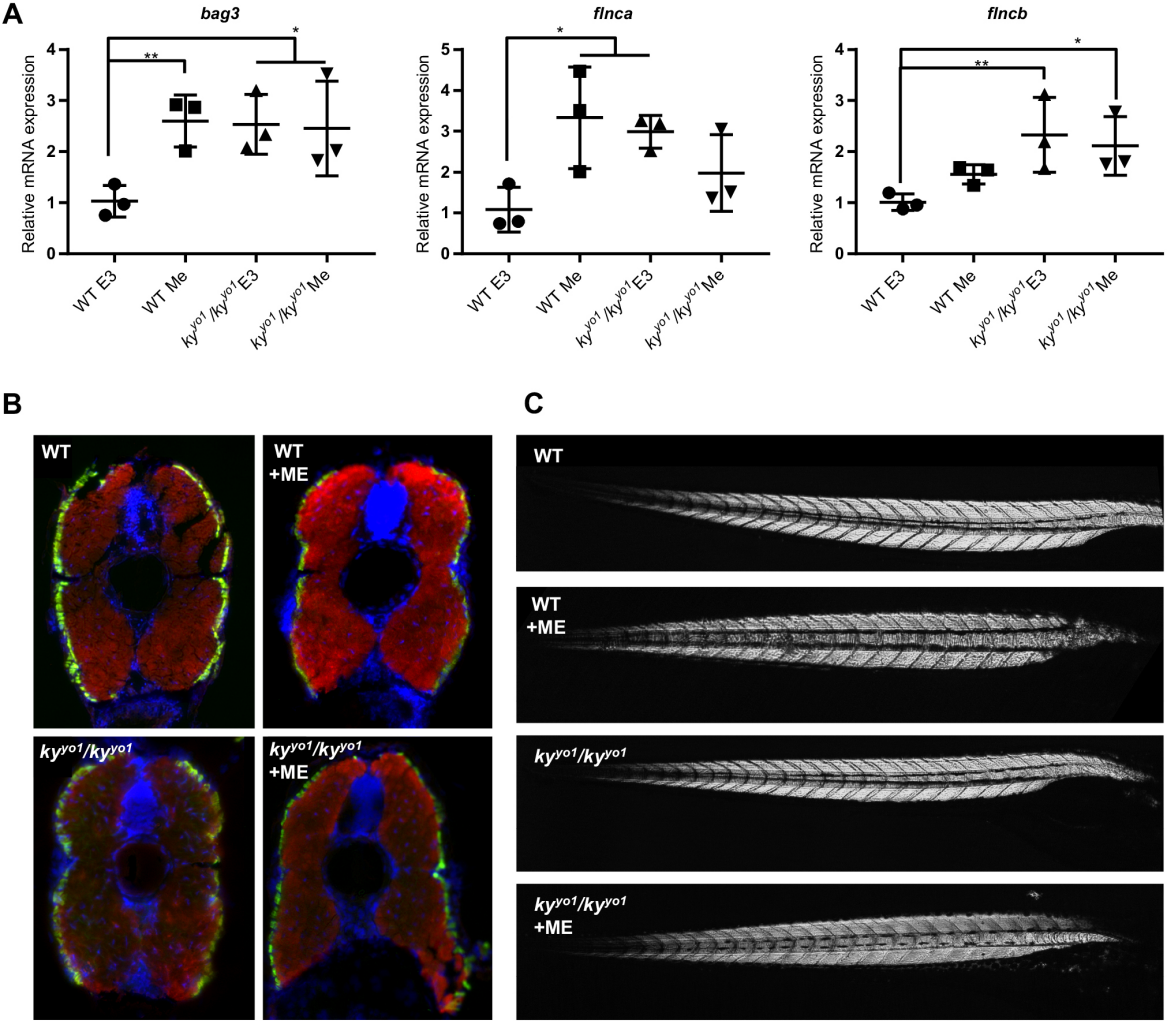
**Methylcellulose challenge does not increase transcriptional upregulation of CASA in *ky^yo1^/ky^yo1^* embryos.** (A) qPCR of *bag3, flnca* and *flncb* shows significantly increased transcription in 5 dpf *ky^yo1^/ky^yo1^* embryos compared with WT controls when grown in normal (E3) media (one-way ANOVA/Dunnett's; *P<0.05, **P<0.01, *n*=3). Swimming in media enriched with 1% methylcellulose (Me) from 3 dpf to 5 dpf induces significant increases in *bag3* and *flnca* transcription and reveals a trend towards increased *flncb* transcription in WT embryos (one-way ANOVA/Dunnett's; *P<0.05, **P<0.01, *n*=3). No significant changes in transcription are observed between challenged and unchallenged *ky^yo1^/ky^yo1^* embryos. Error bars indicate s.e.m. (B) Fibre-typing of embryonic zebrafish muscle shows no changes to slow (green) or fast (red) muscle distribution. Images are representative of five WT and four *ky^yo1^/ky^yo1^* embryos per treatment group (see Fig. S4 for full panel). (C) Representative images of birefringence analysis of whole zebrafish embryos, showing no gross muscle damage or deformation in any treatment group. These observations indicate that transcriptional changes are not secondary to gross muscle damage.

To increase mechanical tension, 3 dpf embryos were moved into E3 media supplemented with 1% methylcellulose, which increases viscosity and makes it more difficult for the fish to swim. Compared with unmodified E3 media, WT embryos show a significant increase in *bag3* and *flnca* expression when raised in viscous media, demonstrating a robust induction of a stress response involving CASA components using this method (Fig. 5A). The *ky^yo1^*/*ky^yo1^* embryos show a significantly higher baseline of *bag3, flnca* and *flncb* expression than WT when swimming in normal media and no further upregulation when raised in viscous media (Fig. 5A). These findings suggest that the Ky deficiency induces a constitutive stress response in zebrafish larvae and reveals an inability of *ky^yo1^*/*ky^yo1^* muscle to further adapt cellular stress responses to the challenge of additional mechanical load. Examination of transverse muscle sections shows no difference in fast/slow muscle distribution between treatment groups, with slow muscle remaining as a peripheral cell layer (Fig. 5B). This observation indicates that transcriptional changes cannot be accounted for by a shift in muscle-fibre type. Additionally, birefringence analysis shows no evidence of gross muscle damage or deformation in any treatment group (Fig. 5C); thus, these transcriptional changes, and the inability of *ky^yo1^/ky^yo1^* embryos to further upregulate CASA components, are not secondary to gross structural damage.

### BAG3 turnover in the *ky/ky* mouse

As the *ky/ky* mutant mouse model demonstrates overt pathology with marked similarity to the human myopathy, we sought to examine whether similar evidence of CASA disruption was apparent in this model. The EDL and soleus muscles were selected for these analyses. The exterior digitorum longus (EDL) is relatively non-pathological compared with the severely dystrophic soleus (Bridges et al., 1992), allowing a distinction between primary, baseline effects of KY deficiency in the EDL and secondary effects observed at high tension and in the context of muscle pathology in the soleus.

**Fig. 6.**
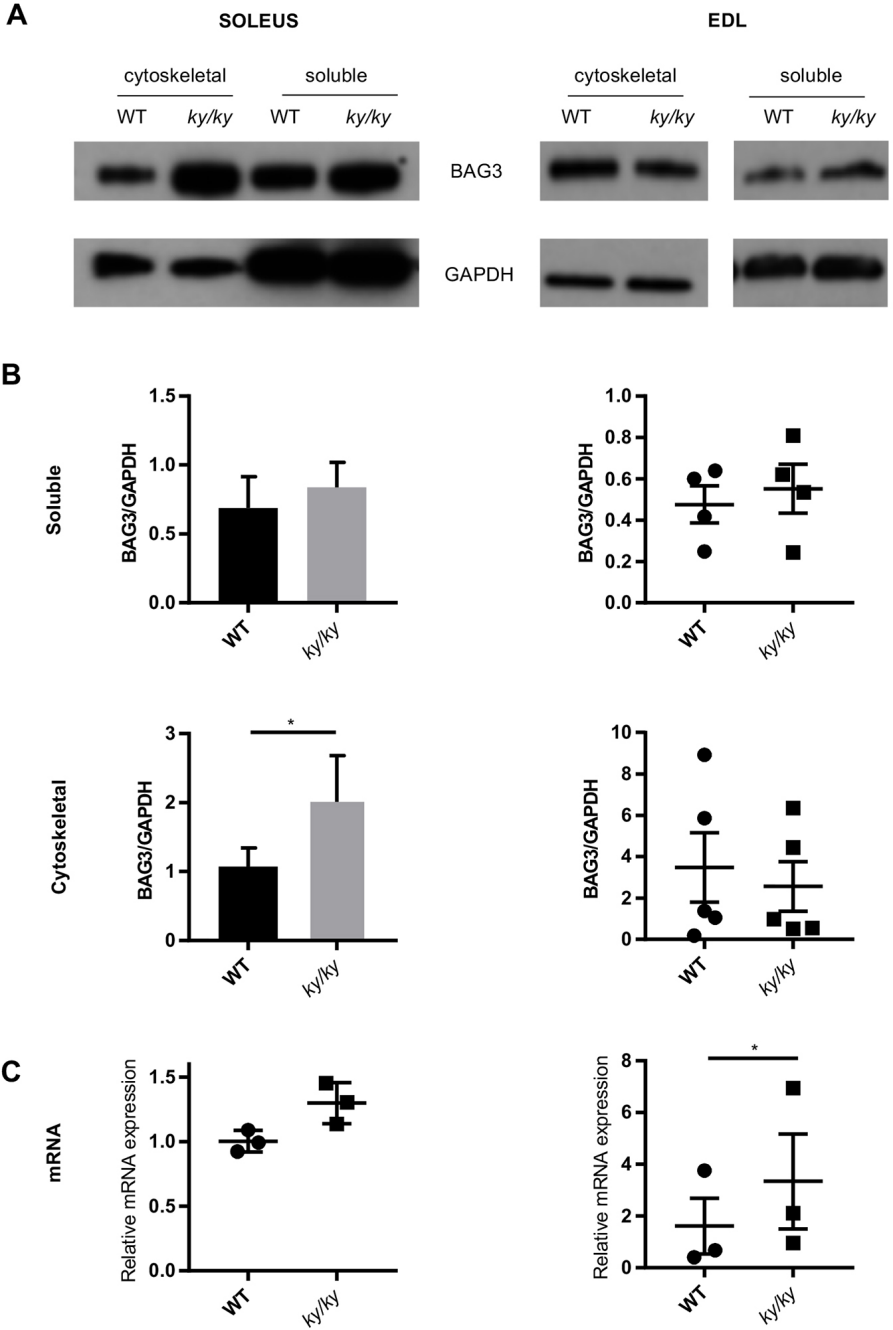
**Altered BAG3 turnover in the *ky/ky* mouse.** (A) Representative western blot images of BAG3 in cytoskeletal and soluble fractions of muscle protein extracted from the soleus and the EDL. (B) Quantification of BAG3 protein levels by densitometry shows significantly higher levels of cytoskeletal-associated BAG3 in the *ky/ky* soleus compared with WT sibling controls (paired Student's *t*-test; *P*<0.05, *n*=8). No differences are observed at the protein level in the EDL. Error bars indicate s.e.m. (C) qPCR of *Bag3* shows no significant difference in transcript levels between WT and *ky/ky* soleus, indicating that the increase in protein might not be accounted for by elevated transcription. Significant upregulation of transcript levels is seen in the EDL (paired Student's *t*-test; *P*<0.05, *n*=3). As no commensurate increase in protein level is observed, this suggests that BAG3 turnover might be elevated in the EDL. Error bars indicate s.e.m.

Initial experiments were unable to detect a difference in BAG3 protein levels between WT and *ky/ky* muscle lysates extracted using RIPA buffer (data not shown). This extraction was only capable of removing soluble proteins, and thus did not allow the detection of elevated amounts of insoluble or cytoskeleton-associated proteins. As impairments to CASA are likely to result in elevated levels of these latter proteins, the total muscle extract sample was processed to obtain a cytoskeletal fraction (see Materials and Methods section for details). In this analysis, elevated levels of cytoskeleton-associated BAG3 were observed in the soleus muscle of *ky/ky* mice compared with WT (Fig. 6 A,B). Consistent with the initial observations using RIPA buffer, there was no significant increase in the amount of soluble BAG3 in the soleus. This finding suggests that an increased proportion of BAG3 protein is associated with the cytoskeleton in the *ky/ky* soleus compared with WT. The non-atrophic EDL showed no indication of elevated soluble or cytoskeletal BAG3 (Fig. 6A,B). Examination of *Bag3* mRNA levels shows a significant increase in the EDL, but not in the soleus (Fig. 6C). This result indicates that the elevated amount of cytoskeletal BAG3 protein in the soleus might not be accounted for by elevated mRNA levels, but is instead indicative of reduced or inefficient turnover. By contrast, the comparable BAG3 protein levels between *ky/ky* and WT EDL (Fig. 6A), combined with elevated mRNA in *ky/ky* EDL (Fig. 6C), indicate that BAG3 turnover is elevated in the mutant EDL tissue.

### Increased immunoreactivity for BAG3 and FLNC in the *ky/ky* soleus

**Fig. 7.**
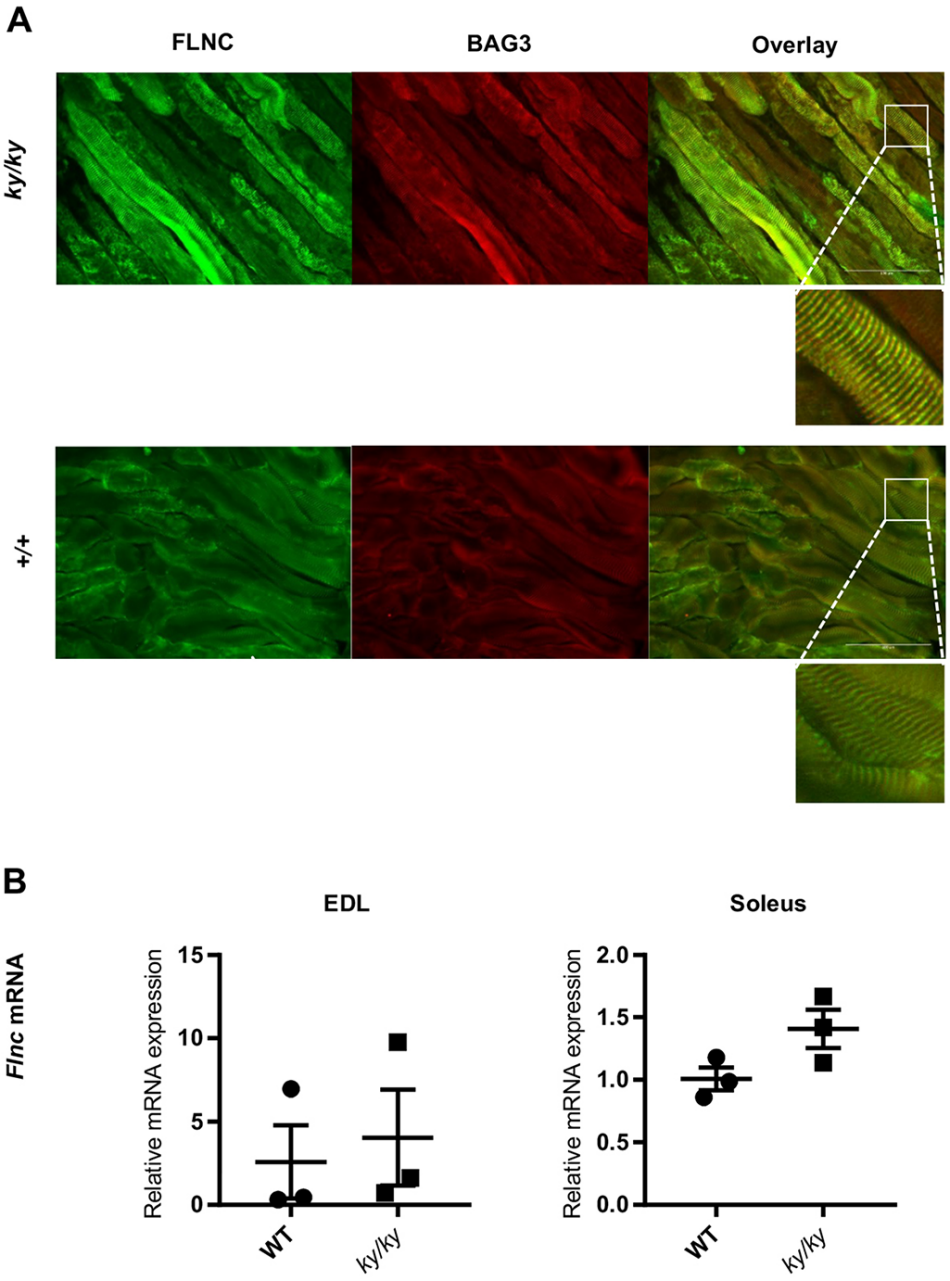
**Increase in BAG3 and FLNC immunoreactivity in *ky/ky* soleus muscle.** (A) Representative images of immunofluorescence against BAG3 and FLNC on longitudinal sections of WT and *ky/ky* soleus. Higher reactivity for BAG3 and FLNC is observed in the *ky/ky* soleus, consistent with the increased cytoskeletal BAG3 observed on western blots in the mutant (see Fig. 6). Amplified insets (squares) show that BAG3 and FLNC appear to remain primarily co-localised to striations, presumed to be the Z-disc. Antibody pools were shared between WT and *ky/ky* sections and camera settings were maintained. (B) qPCR of *Flnc* shows no significant increases when comparing WT and *ky/ky* tissue between siblings (paired student's *t*-test; *P*>0.05, *n*=3). This finding suggests the apparent increase in FLNC protein might not be accounted for by increased transcription. Error bars indicate s.e.m.

Immunofluorescence experiments were performed to visualise the levels and localisation of BAG3 and FLNC in *ky/ky* and WT soleus longitudinal sections. Both BAG3 and FLNC showed increased reactivity in a subset of fibres in the *ky/ky* mutant. Those fibres showing particularly high levels of BAG3 also showed similarly elevated FLNC levels (Fig. 7A). The proteins remained primarily co-localised in a striated pattern, presumably the Z-disc. This observation is consistent with an increased proportion of cytoskeletal-associated BAG3 (Fig. 6A). qPCR was performed to explore whether this increased immunoreactivity represented an increase in *Flnc* transcription. No significant increase in *Flnc* transcript was detected between WT and *ky/ky* tissues (Fig. 7B). The unchanged *Flnc* transcript levels in contrast to the increased reactivity of FLNC antibodies on *ky/ky* soleus sections could be explained by a lower turnover of FLNC protein in the *ky/ky* soleus. This could not be confirmed on western blots with the same FLNC antibodies (RR90), however, owing to inconsistent performance of these antibodies on this application.

## DISCUSSION

Disruptive mutations in the *Ky* gene result in overt muscle pathology in mice (Bridges et al., 1992) and humans (Hedberg-Oldfors et al., 2016; Straussberg et al., 2016; Yogev et al., 2017). Impaired FLNC turnover is a commonly reported molecular phenotype in mice and humans, which potentially implicates CASA in the pathogenic mechanism. In this study we have described the generation of novel C2C12-derived myoblast and zebrafish models of *Ky* deficiency. Significant *Bag3* upregulation was observed in the *ky* homozygous zebrafish at 3 months and in 5 dpf embryos and in the soleus of the *ky/ky* mice. Evidence of CASA upregulation was also apparent in the EDL muscle of the *ky/ky* mice: transcription of *Bag3* was elevated but there was no increase in protein levels, indicating elevated BAG3 turnover.

The disruption to *ky* expression in zebrafish alone was not sufficient to induce overt signs of pathology in adult or embryonic tissue, either under normal conditions or after methylcellulose challenge. Given that KY-deficient pathology in mice and humans occurs primarily in muscles that experience consistent levels of high tension, this disparity might be at least partly explained by the fact that zebrafish muscles do not experience the same gravitational stresses. Unchallenged *ky^yo1^*/*ky^yo1^* embryos showed a transcriptional profile comparable to methylcellulose-treated WT controls, however, suggesting the constitutive transcriptional upregulation of CASA components when deficient in *ky* even in the absence of sustained tension or indicators of structural damage. This phenotype is particularly remarkable considering that the onset of *ky* expression occurs only approximately 48 h prior to this analysis. We cannot rule out that other tests (e.g. electron microscopy or analysis of the expression of embryonic myosin isoform in adult muscle, as observed in human patients; Hedberg-Oldfors et al., 2016) might reveal myopathic phenotypes in the embryonic zebrafish model. Likewise, experiments challenging adult *ky^yo1^*/*ky^yo1^* zebrafish muscle could also reveal myopathic phenotypes.

This lack of overt myopathy in the *ky^yo1^*/*ky^yo1^* model is somewhat mirrored by the fact that the *ky/ky* mouse EDL, a fast-twitch muscle that only experiences tension sporadically, is largely spared by the pathology. This finding suggests that upregulation of CASA components in the zebrafish and spared *ky/ky* mouse EDL, and the trend of upregulation in the myoblast models, represent a primary effect of the absence of KY rather than a downstream consequence of myopathy. By contrast to the EDL muscle, the *ky/ky* mouse soleus experiences consistent high tension and displays the most overt pathology, including atrophy, dystrophic changes and sarcomeric damage (Bridges et al., 1992). The apparent accumulation of cytoskeletal BAG3 with no evident increase in *Bag3* transcription would suggest that in the absence of KY protein turnover in the tonically active soleus is impaired. Accumulation of ubiquitinated proteins in mouse *ky/ky* muscles (Fig. S3) is also consistent with the notion of a role for KY in facilitating protein turnover. Our results do not distinguish whether the absence of KY affects CASA complex solubilisation or that the CASA mechanism is simply overwhelmed by the overarching pathological damage. This phenotype is mirrored by the inability of *ky^yo1^/ky^yo1^* zebrafish embryos to further upregulate *bag3, flnca* and *flncb* in response to mechanical challenge in viscous media, indicating either that the constitutive upregulation of these genes is already maximised, preventing the translation of increased mechanical challenge into this pathway, or that the mechanotransduction mechanism itself is impaired. A role for KY in mechanotransduction has already been proposed on the basis of an inability of *ky/ky* mice to undergo compensatory hypertrophy in response to surgical overloading (Blanco et al., 2001).

Although transcriptional upregulation of CASA components appears to be a consistent early molecular hallmark across the models examined, exactly how this relates to the molecular function of KY and the pathogenic mechanism is unclear. The relationship between autophagy and muscle integrity is complex, with upregulation and downregulation of autophagy contributing to myopathy in different contexts (for a review on autophagy disruption affecting muscle integrity, see Jokl and Blanco, 2016). How much the CASA mechanism contributes overall to skeletal muscle macroautophagy remains uncharacterised, particularly given that FLNC is the only client identified in this context. Examination of macroautophagy flow and signalling in the absence of KY would be highly informative in this regard. Elevated autophagy in *ky/ky* muscles might account for the reduced muscle to body mass ratios observed in *ky/ky* mice, and subsequently the susceptibility of tonically active muscles to structural damage and atrophy. Additionally, BAG3 is proposed to have a role in cytoskeletal stabilisation by facilitating correct localisation of CAPZ, an actin-capping protein (Hishiya et al., 2010). If KY has a function in this process, reduced structural stability in its absence might account for the induction of cell stress.

Our data are consistent with the hypothesis that upregulation of CASA component transcription is a primary compensatory mechanism in response to KY deficiency. This allows low-tension muscles to effectively manage endogenous levels of tension without the emergence of pathology, but is insufficient to meet the demands of high-tension, tonically active muscle. However, further evidence is required to link KY directly to the CASA mechanism or to demonstrate some other function in skeletal muscle maintenance.

## MATERIALS AND METHODS

### Animal research ethics

All animal procedures have been carried out with approval from the University of York Ethics committee and followed the UK Animals (Scientific Procedures) Act 1986 Amendment Regulations 2012, performed under project licenses PPL 70/6827 (mice) and PPL 60/4460 (zebrafish) within an approved establishment (licence 5002510).

### *Ky*-deficient cell-line generation

Px459 plasmid [pSpCas9(BB)-2A-Puro; Addgene] (Ran et al., 2013) was modified by the insertion of annealed, phosphorylated oligonucleotides (Forward: 5′-CACCTCATCGTGCACTCCGAGAAG-3′ and Reverse: 5′-AAACCTTCTCGGAGTGCACGATGA-3′) containing the *Ky* target sequence into the site created by *Age*I digestion of the plasmid. C2C12 myoblasts were transfected with the modified Px459 plasmid using GenJet In Vitro DNA Transfection reagent for C2C12 cells, following the manufacturer's instructions (SL100489-C2C12). To test construct mutagenic capacity, puromycin-resistant cells selected *en masse* were harvested, lysed and the target region amplified by PCR (F: 5′-GGGGCCATTTGCAGCCTA-3′ and R: 5′-CGGAGAGGTTCGGATTAGCC-3′). PCR products were incubated at 37°C for 1 h with T7 endonuclease I; cleavage at the target site indicated successful mutagenesis. Individual clones were isolated by dilution following another round of construct transfection and puromycin selection. Clones were tested for mutagenesis by screening for heteroduplex formation in annealed WT and clone PCR amplicons run on 15% PAGE (Zhu et al., 2014). Alleles were then characterised by TA-cloning of PCR products using a TA Cloning kit (Thermo Scientific) and Sanger sequencing. Clones predicted to have two disruptive alleles (D, I and K) were carried forward for analysis and validated by qPCR (Fig. S1).

### qPCR

For myotubes clones were grown to confluency in 6-well plates before switching to differentiation media [2% serum, 100 U/ml penicillin, 100 μg/ml streptomycin, 0.5 μg/ml fungizone (Gibco) in DMEM]. Cells were allowed to differentiate for 10 days. Progress through differentiation was confirmed visually by light microscopy. Myotubes were washed three times in ice cold phosphate-buffered saline (PBS) before the addition of TRIZOL (Invitrogen) and harvesting by scraping, pooling two wells per biological replicate for a total of three biological replicates. For zebrafish and mice, tissues were snap-frozen and ground under liquid nitrogen before the addition of TRIZOL. The *ky/ky* and control C3H/HeH mice were 7-8 week old males, but not all littermates. Zebrafish embryos were mechanically homogenised in a dounce homogeniser directly in TRIZOL. RNA was extracted using the manufacturer's protocol and converted to cDNA using ReadyScript cDNA synthesis mix (Sigma). qPCR was performed on an ABI StepOnePlus qPCR machine using 2×SYBR Green qPCR master mix (Applied Biosystems). Fold change was calculated using the DDCt method (Livak and Schmittgen, 2001) using either *Hprt* or *Myh7* (mice, C2C12s) or *ef1a* (zebrafish) as the control gene. Mouse primers used were *Hprt* F: 5′-GTTGGATACAGGCCAGACTTTGTT-3′ and R: 5′-GATTCAACTTGCGCTCATCTTAGG-3′ (Cassel et al., 2008); *Flnc* F: 5′-CCTTACTCGCCCTTCCGCATCCAT-3′ and R: 5′-CTCGGGAGCTGTGTAGTAGATGTC-3′ (Chevessier et al., 2015); *Bag3* F: 5′-ATGGACCTGAGCGATCTCA-3′ and R: 5′-CACGGGGATGGGGATGTA-3′ (Rusmini et al., 2015); *Myh7* F: 5′-ACCCTCAGGTGGCTCCGAGA-3′ and R: 5′-TGCAGCCCCAAATGCAGCCA-3′ (Anderson et al., 2015); *Chip/Stub1* F: 5′-CGCAAGGACATTGAGGAGCA-3′ and R: 5′-TAGTCCTCTACCCAGCCGTT-3′; *Hspb8* F: 5′-GCAATGAAATCATCAGCTGGC-3′ and R: 5′-GGGTTGCAGACTTTCTCCAGT-3′; *Ky* F: 5′-ACAGCATGTACCACAAGAGTGAA-3′ and R: 5′-TCTCGATGGTGATTGTGGCTTT-3′. Zebrafish primers used were *ef1a* F: 5′-CTGGAGGCCAGCTCAAACAT-3′ and R: 5′-ATCAAGAAGAGTAGTACCGCTAGCAT-3′ (Tang et al., 2007); *bag3* F: 5′-TGCCCATTCAGATTCAACAG-3′ and R: 5′-GGCTGCTGTGTAGGTTGTTG-3′; *flnca* F: 5′-CCTTCGTGGGTCAGAAGAAC-3′ and R: 5′-GGAGTTCTAGGACCGTGGAC-3′ (Solchenberger et al., 2015); *flncb* F: 5′-GGCCCTACAAAGTGGACATC-3′ and R: 5′-CTTCAAACCAGGCCCATAAG-3′ (Solchenberger et al., 2015); *ky* F: 5′-TGACCCTCATACATCCCAAGC-3′ and R: 5′-GAGCTCCTGTCTGGGGATCA-3′.

### Generation of the *ky^yo1^* zebrafish line

sgRNA targeting the *ky* orthologue was synthesised *in vitro* using an annealed oligonucleotide template (as in Nakayama et al., 2013) with a MEGAshortscript SP6 transcription kit (Ambion) F: 5′-TAATACGACTCACTATAGGGAGGGGTTTCACAATCTCCTCTGTTTTAGAGCTAGAAATAGCAA-3′ and R: 5′-AAAAGCACCGACTCGGTGCCACTTTTTCAAGTTGATAACGGACTAGCCTTATTTTAACTTGCTATTTCTAGCTCTAAAAC-3′. London wild-type (LWT) zebrafish embryos were co-injected with 1 ng recombinant Cas9 protein and 250 pg sgRNA at the 1-16 cell stage into the yolk proximal to the cell body. The *ky* target region from embryos and fin clips was amplified by PCR (F: 5′-AGCCACCAATCAGAAGAAGCA-3′ and R: GTGTTAGCACAGAGTGCACAA-3′) and screened via the high percentage SDS-PAGE assay, as described above, and by Sanger sequencing. This same assay was performed for genotyping in the mutant lines. Off-target screening using *agpat3*, identified as the closest match to the *ky* target sequence by BLAST alignment, showed no evidence of mutagenesis in the same assays from PCR products amplified around the potential off-target site (F: 5′-TGGACCATGATTCAACTGCCC-3′ and R: 5′-AGCTACACTGTTCTGCTCCG-3′). F0 fish were outcrossed to WT to produce heterozygous fish, allowing determination of mutant alleles by subtraction of the WT allele from Sanger sequencing base calls. Heterozygotes carrying the 3 bp deletion or the 5 bp deletion (*ky^yo1^* allele) were incrossed to produce homozygous mutants. For the methylcellulose experiments, incrossing of homozygous mutant fish was performed to produce all *ky^yo1^/ky^yo1^* offspring with a parallel WT incross for the control cohort.

### Western blots

Mice used were 7-8 weeks old. Paired *ky/ky* mice and controls were gender-matched littermates. Protein fractions were isolated from freshly dissected muscle tissue using a Subcellular Protein Fractionation Kit For Tissues (Thermo Scientific; 87790) following the manufacturer's protocol. Samples were combined with 4× NuPAGE LDS buffer (Invitrogen; NP0008) and 10× NuPAGE sample reducing agent (Invitrogen; NP004) and heated to 70°C for 10 min prior to loading onto a 10% Tris-glycine gel. Transfer was performed using the iBlot gel transfer system (Invitrogen) and nitrocellulose stacks (Invitrogen; IB301002). Ponceau S staining was used to check for adequate transfer of proteins to the membrane prior to blocking for 1 h at room temperature (RT) in 5% dried skimmed milk in PBS with Tween 20. Membranes were incubated overnight at 4°C with BAG3 antibody (10599; Proteintech; 1:1000) before three 10 min washes in PBST and incubation with horse radish peroxidase (HRP)-conjugated secondary antibody (anti-rabbit IgG; sc-2030; Santa Cruz Biotechnology, 1:10,000) or HRP-conjugated glyceraldehyde-3-phosphate dehydrogenase (GAPDH; G9295; Sigma; 1:30,000) at RT for 1 h with shaking. Three further washes were performed to remove unbound secondary antibody. The membrane was incubated in Lumisensor HRP substrate (Genscript; L00221V60) for 1 min prior to detection using X-ray film. Levels of protein were determined by band intensity relative to the loading control, as calculated by densitometry using ImageJ software. Blots probed with anti-ubiquitin antibodies (P4D1, Cell Signaling, 1:200) required boiling for 2-5 min for detection of ubiquitinated proteins. P4D1 mouse monoclonal antibody was then detected with a donkey anti-mouse IgG-HRP (Santa Cruz, sc-2314; 1:5000), as described above.

### Methylcellulose challenge

Embryos were raised to 3 dpf in normal E3 media (5 mM NaCl, 0.17 mM KCl, 0.33 mM CaCl_2_, 0.33 mM MgSO_4_ and 0.0001% Methylene Blue), dechorionated and split between unchallenged groups (remaining in normal media) and challenged groups (transferred to media enriched with 1% w/w methylcellulose). Embryos were left for 48 h before harvesting for downstream analyses. Three biological replicates of 15 pooled embryos were used for each treatment.

### Sectioning and histology

Muscle tissue was dissected from sacrificed fish or mice and snap-frozen in liquid N_2_-cooled isopentane and stored at −80°C. A cryostat was used to generate 10 μm sections. For H&E staining of tissue sections, the following protocol was observed: fixation in acetone for 5 s, air drying, 1 min incubation in Gill's Hematoxylin, washing with running water on the back of the slide until clear, one dip in Eosin (no more than 10 s), 20 dips in 100% alcohol (for six alcohol changes), five dips in Histoclear, 30 s incubation in clean Histoclear, and mounting under glass coverslips with DPX mounting media.

### Immunofluorescence

Sections were cut at 12 μm. For mice, control and mutant sections were arranged side-by-side on the same slide to share the same antibody pools. Sections were permeablised and blocked using 3% BSA in PBS+0.3% Triton X100 for 1 h. Sections were incubated for 2 h at RT or overnight at 4°C with primary antibodies against BAG3 (as described previously; 1:200), FLNC (RR90; mouse IgA monoclonal, kind gift from Peter van der Ven, University of Bonn; 1:20), S58 (mouse IgA monoclonal against slow myosin MYH7B, Developmental Studies Hybridoma Bank, DSHB; 1:10), F59 (mouse IgG monoclonal against all fast isoforms, DSHB; 1:10) or ubiquitin (P4D1, a mouse IgG monoclonal, Cell Signaling; 1:200) in blocking buffer followed by three washes with PBS for 5 min. Slides were then incubated for 1 h with appropriate combinations of compatible secondary antibodies at RT in the dark (e.g. anti-Rabbit IgG TRITC; ab6718; 1:70 and anti-Mouse IgA FITC; ab97234; Abcam; 1:70) before three washes with PBS for 5 min. Slides were mounted with Mowiol (Sigma) plus DAPI to stain nuclei. Images were obtained with exposure times of 20 ms (DAPI), 200 ms (FITC) and 300 ms (TRITC), using a Leica DMIL LED microscope plus a Leica DFC 3000 G camera and the Leica LAS X software.

### Statistical analysis

Statistical analysis of data was performed using GraphPad Prism 7 (GraphPad Software). Data were tested for normality using the Shapiro-Wilk test. If normality could be assumed, unpaired two-tailed Student's *t*-tests were used for single comparisons and ANOVA for multiple comparisons with Dunnett's multiple comparison test for post-hoc identification of significantly different means from control. Where normality could not be assumed, the Kruskal-Wallace test was used for multiple comparisons with Dunnett's multiple comparison post-hoc test.

## Supplementary Material

Supplementary information
