# Supplementary material for: Transcriptional upregulation of Bag3, a chaperone-assisted selective autophagy factor, in animal models of KY-deficient hereditary myopathy
Source: Dis Model Mech. 2018 Jul 6;11(7):dmm033225. doi: 10.1242/dmm.033225 (PMC6078408; doi:10.1242/dmm.033225)
Supplement: Supplementary information [file dmm-11-033225-s1.pdf]

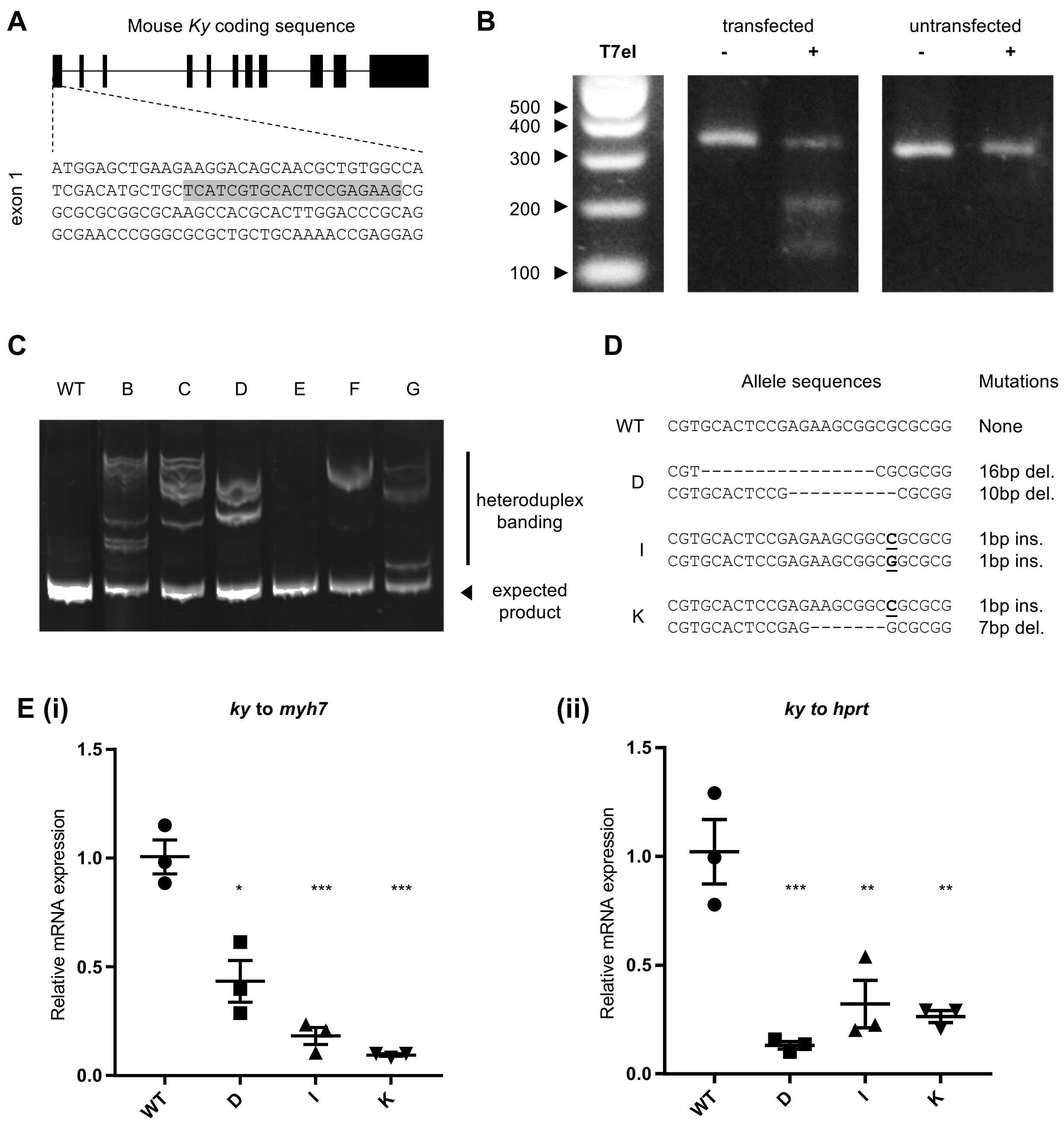

Disease Models & Mechanisms • Supplementary information

Fig S1. Generation and validation of C1C12 derived *Ky* mutant clones.

A) Intron exon structure at scale of the *Ky* gene in mice. Exon 1 sequence is shown, with the CRISPR/Cas9 target sequence highlighted in grey.

B) T7 Endonuclease 1 assay (T7el) of ky PCR product derived from C2C12 cells transfected with the CRISPR/Cas9 *Ky*-targeting plasmid. Note that the predicted products (~140bp + 210 bp) are only obtained in the T7 endonuclease I treated sample from transfected cells, indicating the presence of mutations at the targeting site.

C) Heteroduplex assay of C2C12 targeted clones. Representative gel showing identification of clones carrying mutagenised alleles by the formation of heteroduplex banding. *Ky* PCR products from targeted clones (labelled B to G) and wild type cells (WT) were melted and annealed together and run in high percentage PAGE gels. The presence of additional bands to the expected product (arrow) indicates sequence variations between the *Ky* amplicons in the mix.

D) *Ky* allele sequences of C2C12 derived clones D, I and K. As indicated on the figure, these three clones showed disruptive mutations on both alleles and were therefore selected for further characterisations.

E) Quantitative RT-PCR of *Ky* transcript shows significant disruption to ky expression in differentiated myotubes when normalised to (i) *Myh7* or (ii) *Hprt* (One-way ANOVA/Dunnett's, * $p<0.05$; ** $p<0.01$; *** $p<0.001$, n=3). Error bars represent SEM.

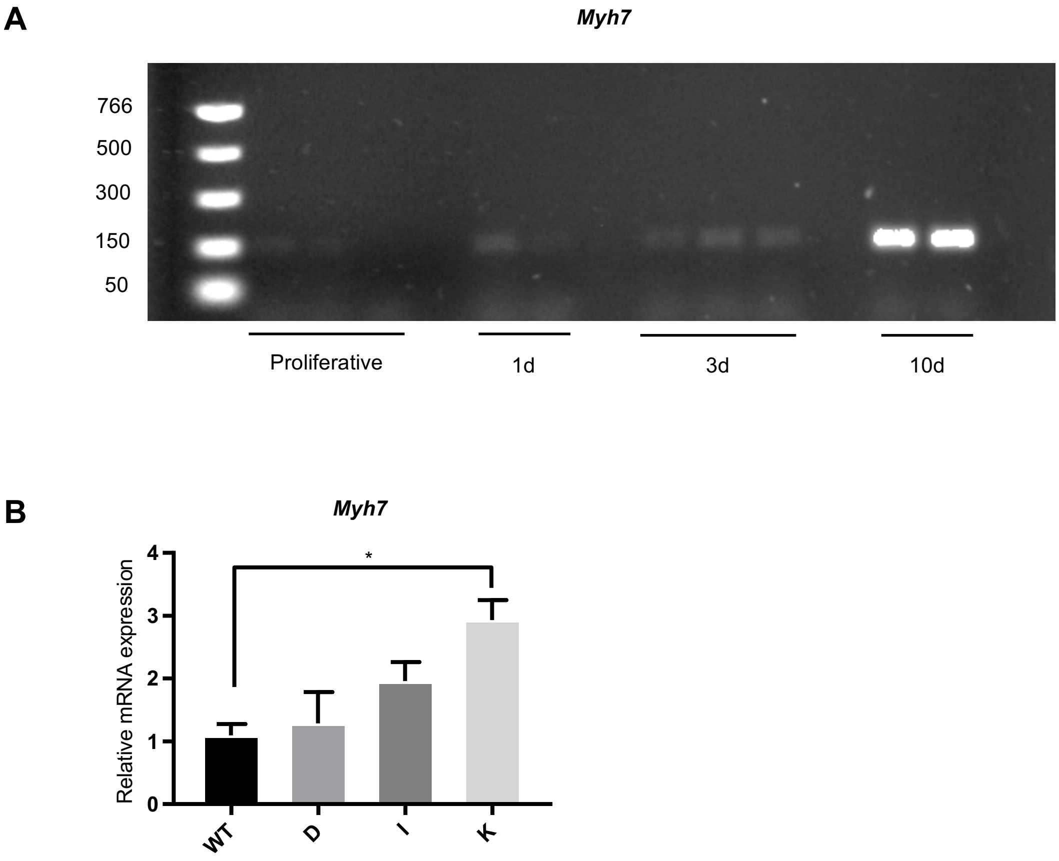

Fig S2. *Myh7* as a marker of myotube differentiation.

A) RT-PCR of *Myh7* shows low levels of expression in proliferative myoblasts and differentiating myoblasts/myotubes after 1 and 3 days in differentiation media. Robust expression is seen after 10 days in differentiation media, indicating *Myh7* is suitable as a marker for late differentiation stages.

B) Quantitative RT-PCR of *Myh7* in WT and disruptive clones shows some variance of transcript levels, though only K is significantly different from WT (One-way ANOVA/Dunnett's, $p<0.05$, n=7). Error bars indicate SEM.

Disease Models & Mechanisms • Supplementary information

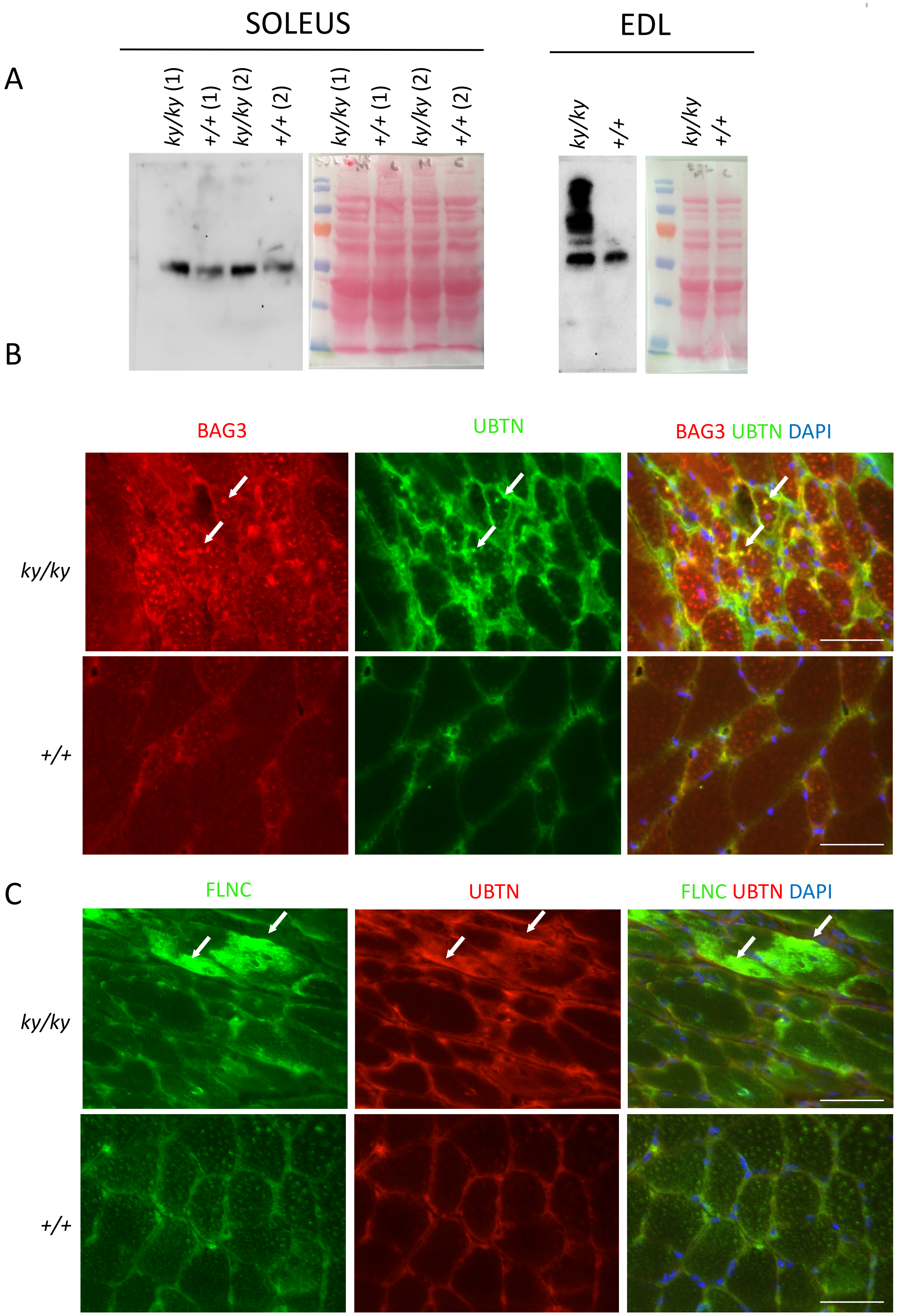
SOLEUS
EDL
A
ky/ky (1)
+/+ (1)
ky/ky (2)
+/+ (2)
ky/ky (1)
+/+ (1)
ky/ky (2)
+/+ (2)
ky/ky
+/+
ky/ky
+/+
B
BAG3
UBTN
BAG3 UBTN DAPI
ky/ky
+/+
C
FLNC
UBTN
FLNC UBTN DAPI
ky/ky
+/+

Fig S3. Endogenous levels of ubiquitinated proteins are higher in muscles from *ky/ky* mice.

A) Western blots of soluble muscle extracts with anti-ubiquitin antibody, as labelled. Samples of soleus were loaded at 40ug. Samples of EDL muscle were loaded at 20ug. Ponceau red stain of the blots indicates equal loading. Each lane indicates the genotype of the mouse, with the number in brackets indicating age and sex matching. B) Immunofluorescence of soleus muscle sections showing higher levels of BAG3 and ubiquitinated proteins in sections from *ky/ky* mice. Images are representative of observations from n=3 mice per genotype. FITC (green) or TRITC (red)- conjugated secondary antibodies were used to detect the primary antibody as indicated by the colour of the labels on each panel. The overlay includes DAPI stained nuclei. White arrows point at regions of overlapping signal in the green and red channels, potentially indicating co-localization of the target proteins. Sections of mutant and control shared the same antibody solution and all images were taken with identical exposure settings. Size bar=50microns. C) Immunofluorescence of soleus muscle sections showing higher levels of FLNC and ubiquitinated proteins in sections from *ky/ky* mice. Images are representative of observations from n=3 mice per genotype. FITC (green) or TRITC (red)- conjugated secondary antibodies were used to detect the primary antibody as indicated by the colour of the labels on each panel. The overlay includes DAPI stained nuclei. White arrows point at regions of overlapping signal in the green and red channels, potentially indicating co-localization of the target proteins. Sections of mutant and control shared the same antibody solution and all images were taken with identical exposure settings. Size bar=50microns.

Disease Models & Mechanisms • Supplementary information

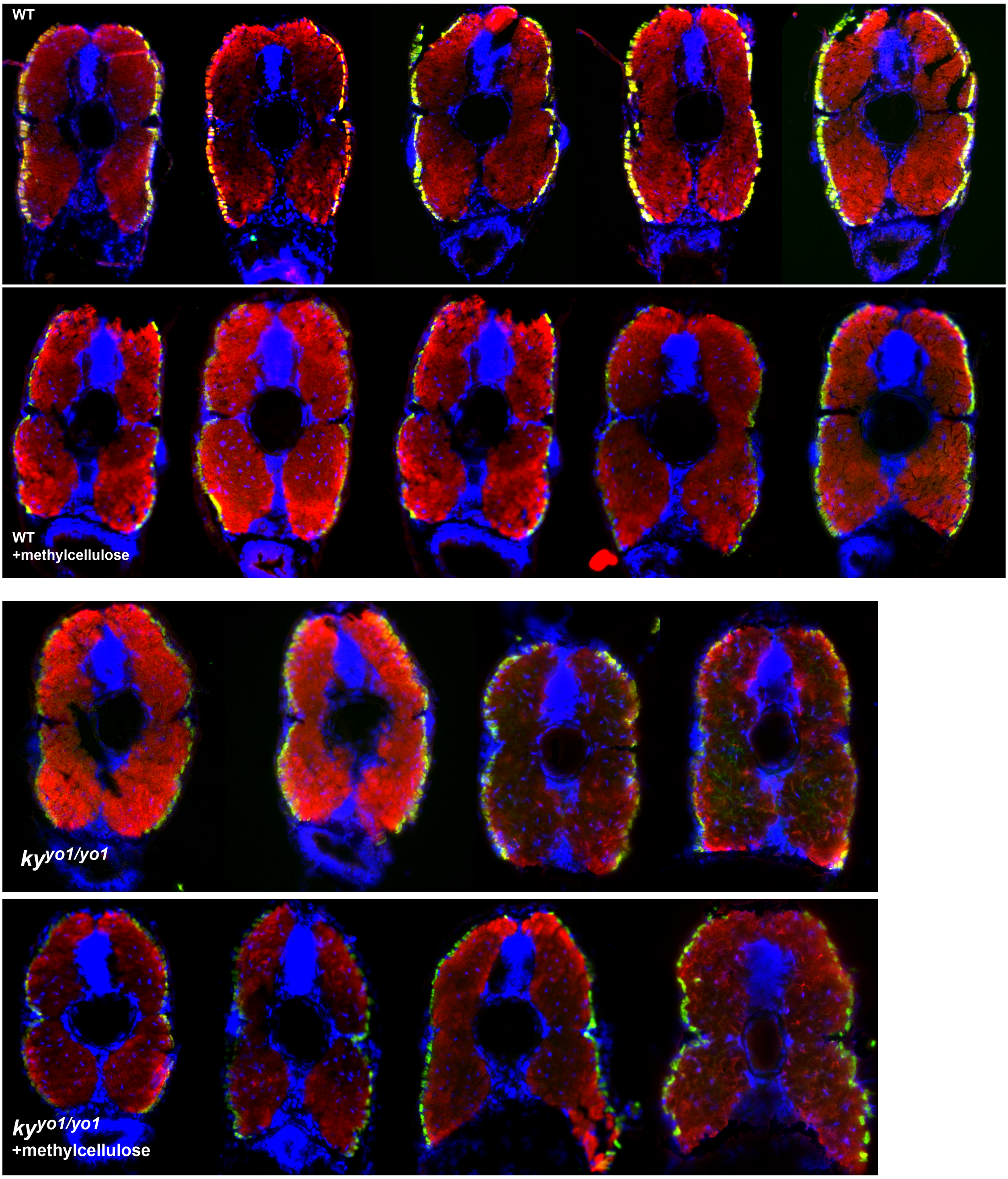

Fig S4. Neither *ky* deficiency or methylcellulose challenge induces changes in fast/slow muscle distribution in zebrafish embryos.

Full panel of sections represented in figure 6C. Fibre-typing of transverse sections of embryonic zebrafish muscle shows no changes to slow (green) or fast (red) muscle distribution in any of the treatment groups (WT/ WT + methylcellulose/ *ky*^{yo1}/*ky*^{yo1} / *ky*^{yo1}/*ky*^{yo1} + methylcellulose), indicating shifts in flnc isoform expression cannot be explained by fibre-type shifting.
